# Reasonable Design of MXene-Supported Dual-Atom Catalysts with High Catalytic Activity for Hydrogen Evolution and Oxygen Evolution Reaction: A First-Principles Investigation

**DOI:** 10.3390/ma16041457

**Published:** 2023-02-09

**Authors:** Erpeng Wang, Miaoqi Guo, Jian Zhou, Zhimei Sun

**Affiliations:** School of Materials Science and Engineering, Beihang University, Beijing 100191, China

**Keywords:** first-principles calculation, dual-atom catalysts (DACs), hydrogen evolution reaction, oxygen evolution reaction, electrocatalyist

## Abstract

MXene-supported single-atom catalysts (SACs) for water splitting has attracted extensive attention. However, the easy aggregation of individual metal atoms used as catalytic active centers usually leads to the relatively low loading of synthetic SACs, which limits the development and application of SACs. Herein, by performing first-principles calculations for Pt and 3d transition metal single atoms immobilized on a two-dimensional (2D) Mo_2_TiC_2_O_2_ MXene surface, we systematically studied the performance of heterogeneous dual-atom catalysts (h-DACs) in hydrogen evolution reaction (HER) and oxygen evolution reaction (OER). Significantly, h-DACs exhibit higher metal atom loading and more flexible active sites compared to SACs. Benefiting from these features, we found that Pt/Cu@Mo_2_TiC_2_O_2_ heterogeneous DACs exhibits excellent HER activity with ultra-low overpotential |ΔG_H_^∗^| (0.04 eV), lower than the corresponding Pt@Mo_2_TiC_2_O_2_ (0.14 eV) and Cu@Mo_2_TiC_2_O_2_ (0.33 eV) SACs, and even lower than that of Pt (0.09 eV). Meanwhile, Pt/Ni@Mo_2_TiC_2_O_2_ exhibits superior OER activity with ultra-low overpotential η^OER^ (0.38 V), lower than that of Pt@Mo_2_TiC_2_O_2_ (1.11 V) and Ni@Mo_2_TiC_2_O_2_ (0.57 V) SACs, and even lower than that of RuO_2_ (0.42 V) and IrO_2_ (0.56 V). Our finding paves the way for the rational design of h-DACs for HER and OER with excellent activity, which provides guidance for other catalytic reactions.

## 1. Introduction

Water splitting technology is debated as the most prospective and sustainable way to produce hydrogen and oxygen, which involves hydrogen evolution reaction (HER) and oxygen evolution reaction (OER) [1,2,3,4,5,6,7,8]. Up to now, the expensive noble metals or their oxides are still deemed as the best choice to propel these reactions, where Pt is used for HER and RuO_2_/IrO_2_ is used for OER [9,10,11]. However, the expensiveness and poor durability restrict their development and commercial applications. Hence, it is of great importance and imminent to explore non-noble metal catalysts with high activity and stability to make the water splitting reaction economical and energy-saving.

Since MXenes was discovered in 2011 [12], a new family of two-dimensional (2D) material rich in early-transition metal carbides, nitrides, or carbonitrides, it has attracted enormous interest in the field of electrocatalysis due to its large specific surface area, high stability, and excellent electronic and thermal conductivity [13,14,15,16,17,18,19,20,21]. MXenes can generally be synthesized by selective removal of the layer of A elements from its parent phase MAX using various methods. Its general chemical formula can be written as M_n+1_AX_n_, where M represents the early transition metals, A is the elements of the group IIIA or IVA, and X is the carbon and/or nitrogen atoms. Numerous experiments and theoretical calculations have confirmed that the outer layers of the nanosheet MXenes are usually terminated by several kinds of functional groups, such as OH, O, or F [22,23,24,25,26]. Surface functional groups can improve the stability of the MXenes substrate, effectively prevent the inner metal layers from being oxidized, and even ameliorate the interaction strength between the MXenes substrate and hydrogen, thus increasing HER activity. However, the functional groups greatly weaken the adsorption strength with OER intermediates (OH^∗^, O^∗^, and OOH^∗^), which is unfavorable for OER [27,28].

Single-atom catalysts (SACs) have received extensive attention in electrocatalysis in the past few years, owing to their fully exposed active sites, maximum atom utilization, extraordinary reaction selectivity, and adjustable surface electronic structure [29,30,31,32,33,34]. Particularly, it has been well confirmed that the SACs composed of a single metal atom anchored on MXenes substrate have shown great application potential in both HER and OER [2,28,35]. In these electrochemical reactions, the single metal atom mainly plays two roles, acting as the active center and acting as a cocatalyst to regulate the local surface electronic structures. However, due to the significantly increased surface free energy of SACs, the single atom embedded on the substrates would spontaneously aggregate to form nanoclusters in the preparation and catalytic process, which greatly reduces catalytic active sites. This shortcoming results in relatively low-density single-atom loading in the currently reported synthesized SACs, generally lower than 1.5 wt% [36,37,38,39,40]. Therefore, it is of great significance to increase the loading capacity of single atoms while maintaining the unique properties of SACs.

Heterogeneous double-atom catalysis (h-DACs) containing two different metal atoms overcomes the drawback of low SACs loading. Moreover, owing to the synergistic effect of two metal atoms, a large amount of unsaturated coordination, and more flexible active sites, h-DACs have shown great application prospects in various catalytic reactions, such as the hydrogen evolution reaction, oxygen reduction reaction, and nitrogen reduction reaction [41,42,43,44,45]. However, the underlying mechanism for the synergistic effect between diatoms on h-DACs to enhance the performance of HER and OER is not very clear, which is still an urgent issue to be addressed at present.

Herein, by means of first-principles calculations, we systematically investigated the HER and OER catalytic activities of the h-DACs composed of a noble metal Pt atom and a non-noble transition metal atom (e.g., 3d: Ti, V, Cr, Fe, Co, Ni, Cu, and Zn) supported on the ordered double transition metal MXene (Mo_2_TiC_2_O_2_) substrate, which has been successfully prepared experimentally [46]. We confirmed that the synergistic effect of h-DACs effectively improves the catalytic activity for HER and OER, and gained a deeper understanding of the reaction mechanism. The result showed that Pt/Cu@Mo_2_TiC_2_O_2_ h-DACs exhibits higher catalytic activity than that of the corresponding Pt@Mo_2_TiC_2_O_2_ SACs and Cu@Mo_2_TiC_2_O_2_ SACs. Pt/Ni@Mo_2_TiC_2_O_2_ h-DACs has great potential in OER, and its OER performance was superior to that of Pt@Mo_2_TiC_2_O_2_ SACs and Ni@Mo_2_TiC_2_O_2_ SACs. Our work extends the exploration of SACs to h-DACs and lays a referential foundation for designing excellent OER and HER catalysts.

## 2. Materials and Methods

Spin-polarized density functional theory (DFT) calculations based on first principles were implemented using the Vienna ab initio simulation package code (VASP) [47]. The Perdew-Burke-Ernzerhof (PBE) functional within the generalized gradient approximation (GGA) was applied to describe the electron exchange-correlation interactions [48,49]. The core-valence interactions were described by the projector-augmented plane-wave (PAW) method and the cutoff energy of plane-wave basis was set to be 500 eV in all the computations. The convergence tolerances for residual force and energy on each atom during the structure relaxation were set to 0.01 eV/Å and 10^−5^ eV, respectively. Monkhorst-Pack k-mesh grid settings of 3 × 3 × 1 and 7 × 7 × 1 were used to sample the 2D Brillouin zone for geometry optimizations and electronic structure calculations, respectively. In all DFT calculations, the vacuum layer thickness in the z-direction was set to 25 Å to avoid interlayer interactions and the van der Waals interaction was considered by using the empirical correction DFT-D3 approach [50]. The VASPKIT code was used to postprocess the data calculated by VASP [51].

The Gibbs free energy change (ΔG) of each elementary reaction step of the HER and OER was calculated adopting the computational hydrogen electrode (CHE) method proposed by Nørskov et al. [52]. According to this method, the ΔG of each elementary reaction step can be expressed as

∆G = ∆E + ∆ZPE − T∆S + ∆G_pH_ + ∆G_U_ (1)

where ∆E is the total energy change of the reaction obtained from DFT calculations; ∆ZPE and T∆S are the zero-point energy change and the entropy change, respectively; and T is temperature and was set to 298.15 K (room temperature) in this work. Moreover, ∆G_U_ = −eU and ∆G_pH_ = −k_B_Tln[H^+^] = pH × k_B_Tln10 represent the influence of electrode potential (U) and pH on the ΔG in the electrochemical elementary step, respectively, where e is the elementary charge and k_B_ is the Boltzmann constant. In this work, the values of both U and pH were assumed to be zero.

The overall reaction scheme of HER could be described as

H^+^ (aq) + e^−^ + ∗ → H^∗^ (2)

H^∗^ → 0.5 H_2_ (g) + ∗ (3)

including three parts, namely the initial state H^+^ (aq) + e^−^, the intermediate adsorbed H^∗^, and the final product of 0.5H_2_ (g). Here, ∗ represents the preferable adsorption site of intermediates on the catalyst surface, and g and aq refer to the gas phase and aqueous solution, respectively. In general, the overpotential is used to evaluate the catalytic activity of the catalyst in the electrocatalysis calculations. As for HER, the Gibbs free energy of hydrogen adsorption (|ΔG_H_^∗^|) is usually selected as the overpotential to judge the catalytic activity. In general, the catalysts with |ΔG_H_^∗^| values of less than 0.2 eV are considered to have high HER catalytic activity, and the closer the value of |ΔG_H_^∗^| is to 0, the higher the HER catalytic activity.

In the acidic environment, the overall OER could be expressed in Equation (4), and the elementary reactions are listed in Equation (5) to Equation (8) [53]:

H_2_O (l) → O_2_ (g) + 4H^+^ + 4e^−^ (4)

H_2_O (l) + ∗ → OH^∗^ + H^+^ + e^−^ (5)

OH^∗^ → O^∗^ + H^+^ + e^−^ (6)

H_2_O (l) + O^∗^ → OOH^∗^ + H^+^ + e^−^ (7)

OOH^∗^ → O_2_ (g) + H^+^ + e^−^ (8)

Here, ∗ represents the active site on the catalyst surface, and g and l refer to the gas phase and the liquid phase, respectively.

The free energy changes for the four elementary OER could be expressed as ∆G_1_ = ∆G_OH_^∗^, ∆G_2_ = ∆G_O_^∗^ − ∆G_OH_^∗^, ∆G_3_ = ∆G_OOH_^∗^ − ∆G_O_^∗^, and ∆G_4_ = 4.92 − ∆G_OOH_^∗^.

For the OER, the overpotential can be obtained from the following equation:

η^OER^ = max{∆G_1_, ∆G_2_, ∆G_3_, ∆G_4_}/e − 1.23 (9)

## 3. Results and Discussion

### 3.1. Stability of MXene-Supported Catalysts

**Figure 1:**
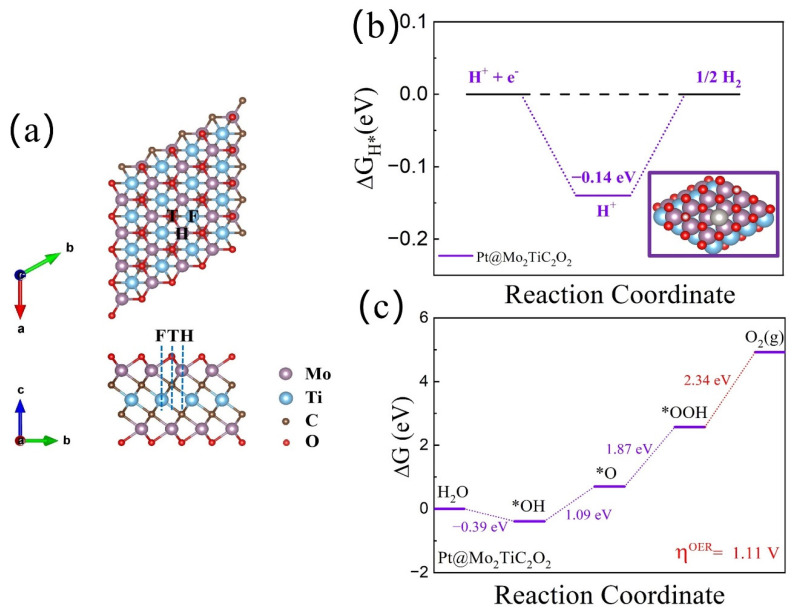
(**a**) Top view and front view of the Mo_2_TiC_2_O_2_ and three different single-atom anchoring sites: fcc (F), top (T), and hcp (H). Calculated free energy profile of Pt@Mo_2_TiC_2_O_2_ SACs for (**b**) HER and (**c**) OER; the red line represents the energy-consuming step. The “∗” symbol represents the adsorption site. a, b and c are the three directional axes of the cell structure, respectively.

First, the adsorption behavior of the single metal Pt embedded on a Mo_2_TiC_2_O_2_ substrate was investigated. As far as we know, the O-terminated Mo_2_TiC_2_ system (denoted as Mo_2_TiC_2_O_2_) has been successfully prepared experimentally [46]. Given the weak interaction between the O-terminal functional groups and the OER reactive intermediates (OH^∗^, O^∗^, and OOH^∗^), it is crucial to embed metal atom active sites on the surface of Mo_2_TiC_2_O_2_ to enhance the ability to activate the reactive intermediates. Based on previous studies, MXene-supported mono-metal Pt catalysts generally exhibit superior HER and OER catalytic activity [53,54,55]. Here, the Pt@Mo_2_TiC_2_O_2_ structure was first constructed by considering three possible Pt atom anchoring sites: fcc (F), top (T), and hcp (H) (as shown in Figure 1). Through DFT calculations, we found that for the three constructed Pt@Mo_2_TiC_2_O_2_ SACs, the top site structure was the most stable, indicating that adsorption is more likely to occur at this site. To evaluate the catalytic activity of Pt@Mo_2_TiC_2_O_2_ SACs for the HER and OER, the Gibbs free energy difference of elementary reaction steps for HER (ΔG_H_^∗^) and OER (ΔG_OH_^∗^, ΔG_O_^∗^, ΔG_OOH_^∗^) processes were calculated separately, as shown in Figure 1b,c. It is not difficult to see that Pt@Mo_2_TiC_2_O_2_ SACs has superior HER catalytic performance with a low |ΔG_H_^∗^| value of 0.14 eV, close to that of Pt (0.09 eV) [11]. However, the catalytic performance of the OER is not as good as expected, showing a larger overpotential barrier of 1.11 V. Then, by simultaneously loading a 3d transition metal atom (TM = Ti, V, Cr, Fe, Co, Ni, Cu, and Zn) on the surface of Pt@Mo_2_TiC_2_O_2_ to adjust the electronic structure, it is expected to effectively improve the catalytic activity of Pt@Mo_2_TiC_2_O_2_ for HER and OER. Here, the constructed structures were called heterogeneous double-atom catalysts (denoted as Pt/TM@Mo_2_TiC_2_O_2_ h-DACs).

**Figure 2:**
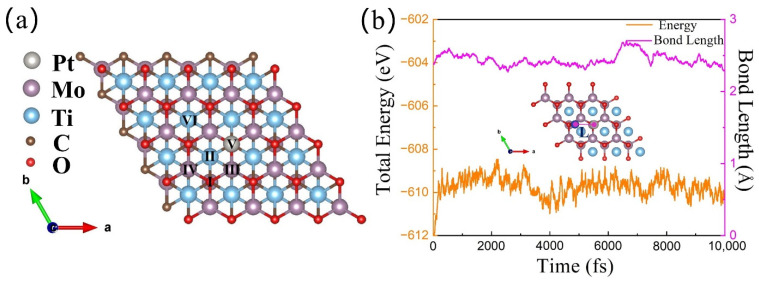
(**a**) The schematic diagrams of the TM atomic possible adsorption sites on the surface of Pt@Mo_2_TiC_2_O_2_ (TM = Sc, Ti, V, Cr, Fe, Co, Ni, Cu, and Zn), i.e., Site I, Site II, Site III, Site IV, Site V, and Site VI. (**b**) The evolution of bond length (L) between Cu and Pt atoms and the total energy for Pt/Cu@Mo_2_TiC_2_O_2_ h-DACs within 10,000 fs in AIMD simulations performed at 300 K. a, b and c are the three directional axes of the cell structure, respectively.

Figure 2 illustrates schematic diagrams of the TM atomic possible adsorption sites on the surface of Pt@Mo_2_TiC_2_O_2_, namely Site I, Site II, Site III, Site IV, Site V, and Site VI. The Pt/TM@Mo_2_TiC_2_O_2_ h-DAC structures with the lowest surface adsorption energies are depicted in Figure S1. Talking the Pt/Cu@Mo_2_TiC_2_O_2_ h-DAC structure as an example, we considered six kinds of possible adsorption sites of the Cu atom, obtained five systems with stable structure after structure optimization, and then selected the configuration with the lowest total energy for subsequent calculations (see Figure S1). In order to further evaluate the dynamic stability of the above selected systems, ab initio molecular dynamics (AIMD) simulations were implemented at 298 K for 10 ps. For the AIMD simulations, the change in bond length (L) between Cu and Pt atoms and the total energy of h-DACs with time were used to verify the thermal stability (as shown Figure 2b). It can be easily observed that the fluctuation of bond length and total energy was small, indicating that the structure shows high thermodynamic stability.

### 3.2. HER Catalytic Activity of h-DACs

**Figure 3:**
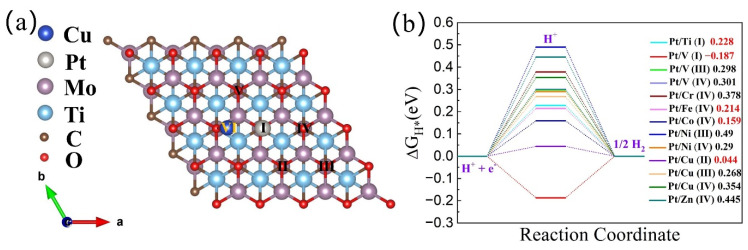
(**a**) Six possible H proton adsorption sites on the Pt/Cu@Mo_2_TiC_2_O_2_ h-DACs’ surface, i.e., Site I, Site II, Site III, Site IV, Site V, and Site VI. (**b**) Calculated Gibbs free energy profiles of HER at different sites for Pt/TM@Mo_2_TiC_2_O_2_ h-DACs (TM = Ti, V, Cr, Fe, Co, Ni, Cu, and Zn). The red numbers represent the catalysts with high HER catalytic activity. a, b and c are the three directional axes of the cell structure, respectively.

We first investigated the catalytic activity of Pt/TM@Mo_2_TiC_2_O_2_ h-DACs for the HER. Similarly, adequate testing was also required to determine the stable adsorption site of H protons on the h-DACs’ surface. For h-DACs, H protons generally tend to be adsorbed on the upper end of O or embedded transition metals, where the embedded transition metal atoms play the role of cocatalyst and catalyst, respectively. Here, Pt/Cu@Mo_2_TiC_2_O_2_ h-DACs were also taken as an example to illustrate. As displayed in Figure 3, there are six possible H adsorption sites on the Pt/Cu@Mo_2_TiC_2_O_2_ h-DACs’ surface, namely Site I, Site II, Site III, Site IV, Site V, and Site VI. The possible adsorption sites of H protons in other studied h-DAC systems are shown in Figure S2. Then, the HER catalytic activity calculations were conducted on these sites. The calculated free energy for H^∗^ adsorption (ΔG_H_^∗^) of all the studied h-DACs at different active sites are presented in Table S1 and Figure 3b. It can be seen from Figure 3b that the |ΔG_H_^∗^| values of Pt/Cu@Mo_2_TiC_2_O_2_ h-DACs at Site II, Pt/Co@Mo_2_TiC_2_O_2_ h-DACs at Site IV, and Pt/V@Mo_2_TiC_2_O_2_ h-DACs at Site I are less than 0.2 eV, manifesting excellent HER catalytic activity. It should be noted that the Pt and Cu atoms on Pt/Cu@Mo_2_TiC_2_O_2_ (Site II) and Pt and Co atoms on Pt/Co@Mo_2_TiC_2_O_2_ (Site IV) are cocatalysts that act as an electron catalytic promoter, while the V atom on Pt/V@Mo_2_TiC_2_O_2_ (Site I) is a catalyst that participates in the catalytic reactions. Particularly, Pt/Cu@Mo_2_TiC_2_O_2_ has an ultra-low |ΔG_H_^∗^| value of 0.04 eV, even better than that of benchmark Pt (0.09 eV), which probably serves as a promising HER catalyst. For comparison, the HER catalytic activities of the corresponding Pt@Mo_2_TiC_2_O_2_ SACs and Cu@Mo_2_TiC_2_O_2_ SACs were also tested. The stable H^∗^ adsorption sites and the corresponding free energy for H^∗^ adsorption (ΔG_H_^∗^) were displayed in Figure S3. The lowest |ΔG_H_^∗^| values of all adsorption sites for Pt@Mo_2_TiC_2_O_2_ (Site III) and Cu@Mo_2_TiC_2_O_2_ (Site IV) are 0.14 eV and 0.33 eV, respectively, significantly higher than 0.04 eV for Pt/Cu@Mo_2_TiC_2_O_2_, which further indicates that the introduction of double atoms improves the HER catalytic activity of the corresponding SACs.

### 3.3. OER Catalytic Activity of h-DACs

**Figure 4:**
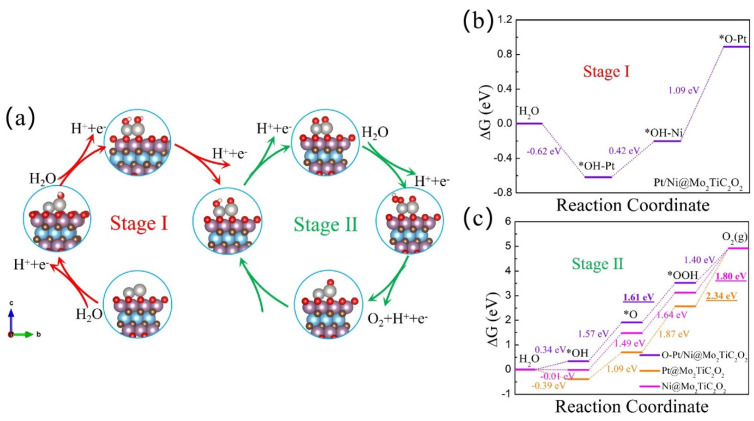
(**a**) The configurations of oxygenated intermediates in energy-favored reaction pathways of OER on Pt/Ni@Mo_2_TiC_2_O_2_ h-DACs. The Gibbs free energy diagrams of reaction mechanisms for (**b**) Stage I and (**c**) Stage II and Pt@Mo_2_TiC_2_O_2_ SACs and Ni@Mo_2_TiC_2_O_2_ SACs. The underlined number represents the most energy-consuming step. The “∗” symbol represents the adsorption site. a, b and c are the three directional axes of the cell structure, respectively.

We then continued to investigate the OER catalytic activity of these Pt/TM@Mo_2_TiC_2_O_2_ h-DACs. The widely accepted entire OER process on SACs can be divided into four stages (see Equations (5)–(8)), which involve three intermediate states (OH^∗^, O^∗^, and OOH^∗^). However, for h-DACs, the OER behavior is more complicated than that of SACs due to the more flexible catalyst active sites. Here, we chose Pt/Ni@Mo_2_TiC_2_O_2_ h-DACs as an example to illustrate in detail. All possible reaction paths occurring on Pt/Ni@Mo_2_TiC_2_O_2_ h-DACs are displayed in Figure S4, where the energy-favored reaction pathways are marked with red lines. The optimal reaction pathways can be summarized as shown in Figure 4a and described as follows: firstly, the OH^∗^ adsorption state was more inclined to spontaneously adsorb to the Pt atom in bare Pt/Ni@Mo_2_TiC_2_O_2_ h-DACs accompanied by a −0.62 eV adsorption free energy change. Then, the second OH^∗^ was then adsorbed to the Ni atom with the adsorption free energy change of 0.42 eV. Subsequently, the OH^∗^ adsorption state on the Ni atom was preferentially transformed into the O^∗^ adsorption state, which requires a 1.09 eV reaction energy and releases proton and electron pairs at the same time. Next, it is interesting to note that the subsequent reactions tend to occur only on the Ni single atom to complete the whole four-electron OER reaction process, with the Gibbs free energies of 1.57 eV, 1.61 eV, 1.40 eV, and 0.34 eV required for each elementary reaction separately. In summary, the entire OER on Pt/Ni@Mo_2_TiC_2_O_2_ h-DACs can be divided into two stages, namely the 3e^−^ reaction occurring on Ni and Pt atoms (Stage I) and the cyclic OER along the 4e^−^ reaction pathways involving only an Ni atom (Stage II). The OER reaction pathways of other studied Pt/TM@Mo_2_TiC_2_O_2_ h-DAC systems are displayed in Figures S5–S11, respectively. From Figure 4b,c, for Pt/Ni@Mo_2_TiC_2_O_2_ h-DACs, the formation of OOH^∗^ on the Ni atom is the most energy-consuming step, showing the maximum step distance. Furthermore, we found that Pt/Cr@Mo_2_TiC_2_O_2_ h-DACs and Pt/Ni@Mo_2_TiC_2_O_2_ h-DACs exhibited excellent OER catalytic activity (see Figure 4c and Figure S12), with low overpotentials of 0.49 eV and 0.38 eV, respectively. Especially for Pt/Ni@Mo_2_TiC_2_O_2_ h-DACs, the ultra-low overpotential of 0.38 V was obviously lower than the corresponding data for Pt@Mo_2_TiC_2_O_2_ SACs (1.11 V) and Ni@Mo_2_TiC_2_O_2_ SACs (0.57 V), and even lower than that of RuO_2_ (0.42 V) and IrO_2_ (0.56 V) [10]. Therefore, Pt/Ni@Mo_2_TiC_2_O_2_ h-DACs is promising excellent OER catalysts. Our findings show that the introduction of double atoms can also ameliorate the OER catalytic activity of the corresponding SACs.

**Figure 5:**
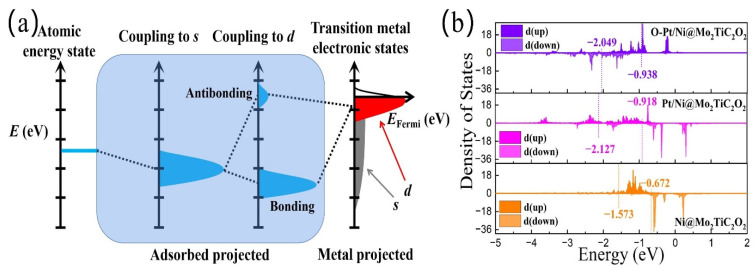
(**a**) The schematic diagram of d-band center theory mechanism. (**b**) The projected DOS and the position of d-band center of Ni atom for Ni@Mo_2_TiC_2_O_2_, Pt/Ni@Mo_2_TiC_2_O_2_, and O-Pt/Ni@Mo_2_TiC_2_O_2_. The position of d-band center of spin-up and spin-down are marked with numbers, respectively.

It is known that good conductivity is the prerequisite for the operation of electrocatalysts, which can be directly observed from its electronic structure. In order to deeply understand the catalytic activity of h-DACs for the OER, we further calculated their electronic density of states (DOS). As shown in Figure S13b, the average DOS per atom states of Pt/Ni@Mo_2_TiC_2_O_2_ h-DACs and Pt/Cr@Mo_2_TiC_2_O_2_ h-DACs with high OER catalytic activity is continuous at the Fermi level, implying that they are metal-conductive and conducive to electron transport. For the Pt/Ni@Mo_2_TiC_2_O_2_ h-DACs, the adsorption of OH^∗^ in the first elementary step and formation of O^∗^ in the second elementary step are the main differences from Ni@Mo_2_TiC_2_O_2_ SACs (as shown in Figure 4c). Therefore, we focus on these differences in the subsequent discussion. It is well known that the total Gibbs free energy change for a complete OER progress is 4.92 eV, so the free energy differences for each elementary step of the most ideal OER catalyst should be 1.23 eV. For Ni@Mo_2_TiC_2_O_2_ SACs, the sum of the Gibbs free energy change of the first two elementary steps is 1.48 eV, which makes the subsequent two elementary steps need to share the free energy difference of 3.44 eV, resulting in a large overpotential for OER. This situation was well ameliorated on the Pt/Ni@Mo_2_TiC_2_O_2_ h-DACs, in which the structure of O-Pt plays an important role. This can be understood from the perspective of d-band center theory. When the adsorbate OH^∗^ binds to the Ni metal atom, the p orbital of the O atom would couple with the d orbital of the Ni atom to form a bonding state and an anti-bonding state (as shown in Figure 5a). The electrons occupying the bonding state make the whole system more stable; on the contrary, the occupation in the anti-bonding state makes the system unstable. The higher (lower) the d-band center of a metal site, the more (less) electrons filled into anti-bonding state, and the stronger (weaker) the affinity with the adsorbate [56]. It can be seen that the synergistic effects of the Pt atom and Ni atom reduce the d-band center of the Ni metal site, especially that the co-adsorbed ^∗^O-Pt/Ni@Mo_2_TiC_2_O_2_ structure further reduces the position of the d-band center of the Ni metal site (See Figure 5b), which leads to a decrease in the adsorption energy bound to the OH adsorbate. Thus, the free energy change in the first two elementary steps of the OER was effectively improved, thereby reducing the OER overpotential and remarkably promoting the catalytic activity. Moreover, this result is consistent with that of the Bader charge transfer on the Ni atom, which decreases from −0.89e of Ni@Mo_2_TiC_2_O_2_ SACs to −0.79e of Pt/Ni@Mo_2_TiC_2_O_2_ h-DACs and further decreases to −0.76e of ^∗^O-Pt/Ni@Mo_2_TiC_2_O_2_ h-DACs (See Table S2).

## 4. Conclusions

In conclusion, on the basis of the DFT first-principles calculations, we systematically explored the OER and HER catalytic activity of Pt/TM@Mo_2_TiC_2_O_2_ heterogeneous double-atom catalysis (TM = Ti, V, Cr, Fe, Co, Ni, Cu, and Zn). Our computations demonstrated that the constructed h-DACs catalysts have more flexible activity centers than the SACs and the h-DACs, which significantly increase the loading of metal atoms. In addition, the synergistic effect of bimetallic atoms of h-DACs enhances the OER and HER catalytic activity of the corresponding SACs. We found that Pt/Cu@Mo_2_TiC_2_O_2_, Pt/V@Mo_2_TiC_2_O_2_, and Pt/Co@Mo_2_TiC_2_O_2_ h-DACs showed great potential in HER catalysis and Pt/Ni@Mo_2_TiC_2_O_2_ and Pt/Cr@Mo_2_TiC_2_O_2_ h-DACs exhibited superior OER catalytic activity. In particular, two h-DACs, Pt/Cu@Mo_2_TiC_2_O_2_ and Pt/Ni@Mo_2_TiC_2_O_2_, exhibited ultra-low overpotentials for the HER (|ΔG_H_^∗^| = 0.04 eV) and OER (η^OER^ = 0.38 V), respectively, which are even better than those of the benchmark Pt (|ΔG_H_^∗^| = 0.09 eV) and RuO_2_ (η^OER^ = 0.42 V) or IrO_2_ (η^OER^ = 0.56 V). Specifically, the formation of the co-adsorbed ^∗^O-Pt/Ni@Mo_2_TiC_2_O_2_ structure clarifies the origin of the high catalytic activity of Pt/Ni@Mo_2_TiC_2_O_2_ h-DACs. Our findings provide a valuable reference for the rational design of catalysts with promising applications for OER and HER.

## Data Availability

This data presented in this study are available on request from the corresponding authors.

## Supplementary Materials

The following supporting information are available online at https://www.mdpi.com/article/10.3390/ma16041457/s1, Figure S1: The most stable structures diagram for Pt/TM@Mo_2_TiC_2_O_2_ h-DACs (TM = Ti, V, Cr, Fe, Co, Ni, Cu and Zn), Figure S2. The possible H proton adsorption sites on the (a) Pt/TM@Mo_2_TiC_2_O_2_ h-DACs surface (TM = Ti, V, Cr and Fe); (b) Pt/Co@Mo_2_TiC_2_O_2_ h-DACs surface; (c) Pt/Ni@Mo_2_TiC_2_O_2_ h-DACs surface; (d) Pt/TM@Mo_2_TiC_2_O_2_ h-DACs surface (TM = Cu, Zn), Figure S3. The possible H proton adsorption sites on the (a) Pt@Mo_2_TiC_2_O_2_ SACs surface and (b) Cu@Mo_2_TiC_2_O_2_ SACs. (c) Calculated Gibbs free energy profiles of HER for Pt@Mo_2_TiC_2_O_2_ SACs, Cu@Mo_2_TiC_2_O_2_ SACs and Pt/Cu@Mo_2_TiC_2_O_2_ h-DACs, Figure S4. The diagram of all possible ORR pathways and corresponding Gibbs free energy on Pt/Ni@Mo_2_TiC_2_O_2_ h-DACs, red lines represent the energy-favored reaction pathways, Figure S5. The diagram of all possible ORR pathways and corresponding Gibbs free energy on Pt/Ti@Mo_2_TiC_2_O_2_ h-DACs, red lines represent the energy-favored reaction pathways, Figure S6. The diagram of all possible ORR pathways and corresponding Gibbs free energy on Pt/V@Mo_2_TiC_2_O_2_ h-DACs, red lines represent the energy-favored reaction pathways, Figure S7. The diagram of all possible ORR pathways and corresponding Gibbs free energy on Pt/Cr@Mo_2_TiC_2_O_2_ h-DACs, red lines represent the energy-favored reaction pathways, Figure S8. The diagram of all possible ORR pathways and corresponding Gibbs free energy on Pt/Fe@Mo_2_TiC_2_O_2_ h-DACs, red lines represent the energy-favored reaction pathways, Figure S9. The diagram of all possible ORR pathways and corresponding Gibbs free energy on Pt/Co@Mo_2_TiC_2_O_2_ h-DACs, red lines represent the energy-favored reaction pathways, Figure S10. The diagram of all possible ORR pathways and corresponding Gibbs free energy on Pt/Cu@Mo_2_TiC_2_O_2_ h-DACs, red lines represent the energy-favored reaction pathways, Figure S11. The diagram of all possible ORR pathways and corresponding Gibbs free energy on Pt/Zn@Mo_2_TiC_2_O_2_ h-DACs, red lines represent the energy-favored reaction pathways, Figure S12. The configurations of oxygenated intermediates in energy-favored reactions pathways of OER on Pt/Cr@Mo_2_TiC_2_O_2_ h-DACs. The red line represents the most energy-consuming step, Figure S13. (a) The Projected DOS and the location d-band center of Pt atom for Pt@Mo_2_TiC_2_O_2_, Pt/Cr@Mo_2_TiC_2_O_2_ and Pt-O-Cr@Mo_2_TiC_2_O_2_ h-DACs. (b) The electronic DOS (per atom) of Pt/Cr@Mo_2_TiC_2_O_2_ h-DACs and Pt/Ni@Mo_2_TiC_2_O_2_ h-DACs, Table S1. ΔGH* of different H adsorption sites on the Pt/TM@Mo_2_TiC_2_O_2_ DACs surface (TM = Ti, V, Cr, Fe, Co, Ni, Cu and Zn). Wherein, “x” represents structural instability after H proton adsorbed to the site. “-” represents the non-existent sites, Table S2. The number of the charge transfer (Qe, e) from Sigle-atom to substrate.
