# Supplementary material for: Reasonable Design of MXene-Supported Dual-Atom Catalysts with High Catalytic Activity for Hydrogen Evolution and Oxygen Evolution Reaction: A First-Principles Investigation
Source: Materials (Basel). 2023 Feb 9;16(4):1457. doi: 10.3390/ma16041457 (PMC9958578; doi:10.3390/ma16041457)
Supplement: Supplementary file 1 [file materials-16-01457-s001.zip › materials-2163512-supplementary.pdf]

materials

MDPI

Supplementary Materials

Reasonable Design of MXene-Supported Dual-Atom Catalysts with High Catalytic Activity for Hydrogen Evolution and Oxygen Evolution Reaction: A First-Principles Investigation

Erpeng Wang, Miaoqi Guo, Jian Zhou * and Zhimei Sun *

School of Materials Science and Engineering, Beihang University, Beijing 100191, China
* Correspondence: jzhou@buaa.edu.cn (J.Z.); zmsun@buaa.edu.cn (Z.S.)

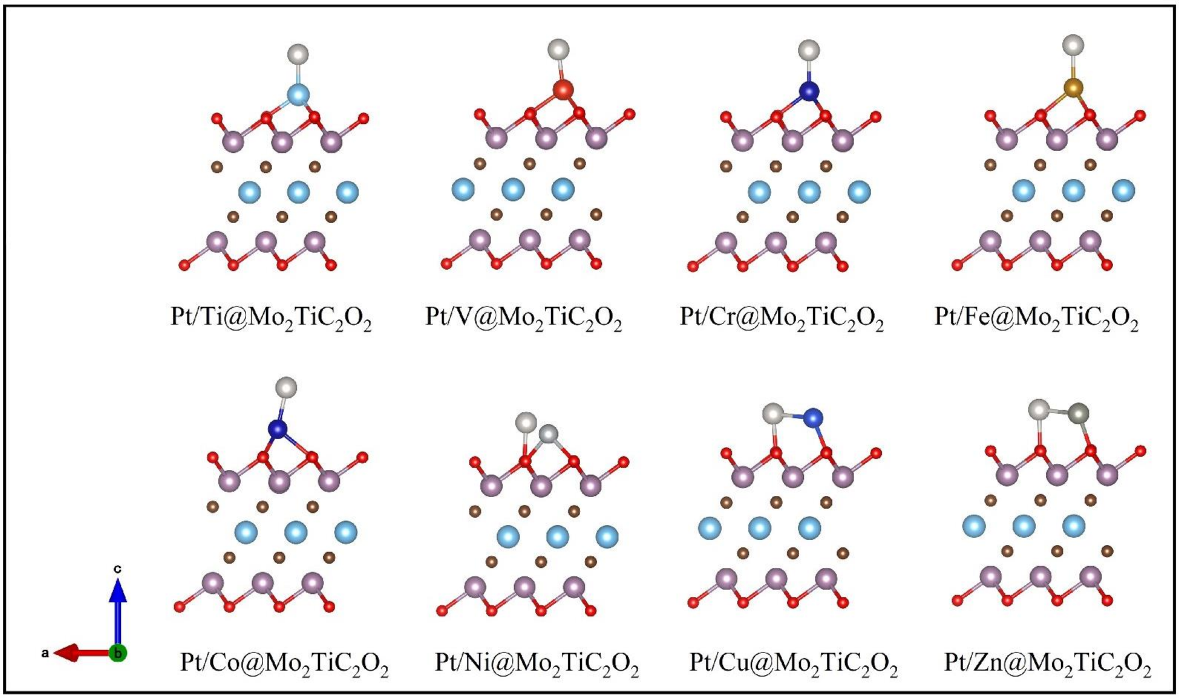

Figure S1. The most stable structures diagram for Pt/TM@$Mo_2TiC_2O_2$ h-DACs (TM = Ti, V, Cr, Fe, Co, Ni, Cu and Zn).

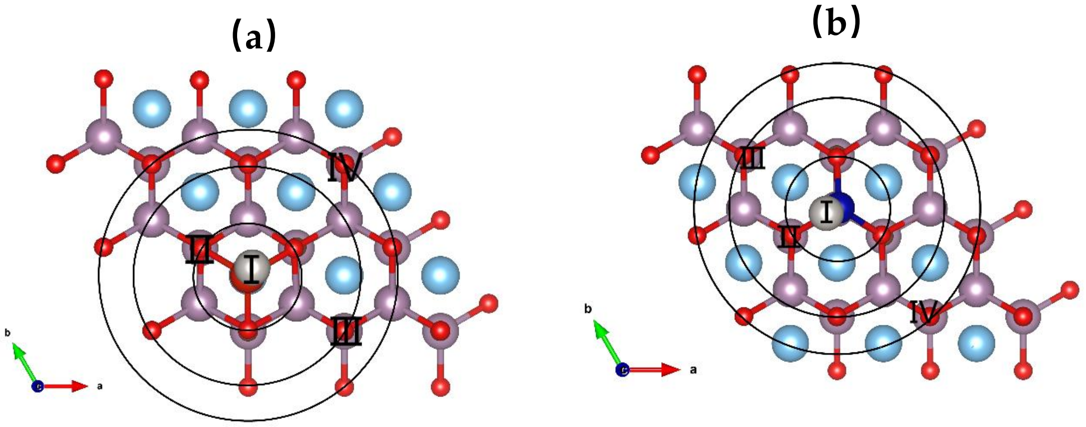

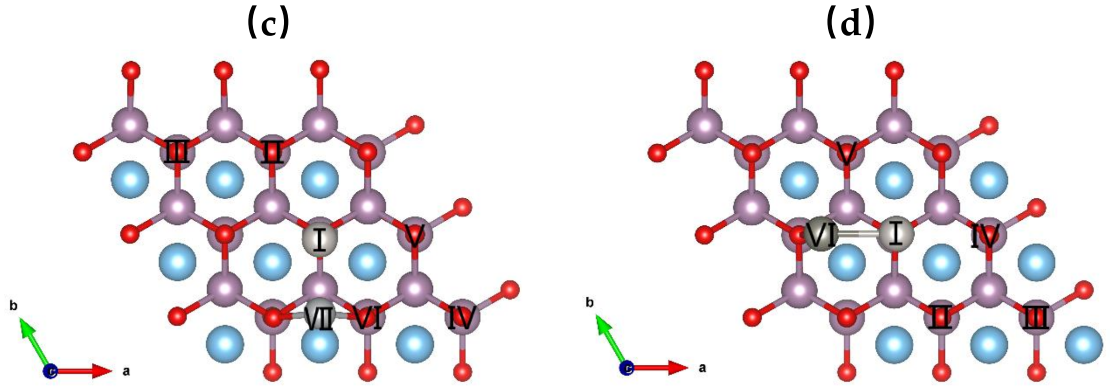

Figure S2. The possible H proton adsorption sites on the (a) Pt/TM@$Mo_2TiC_2O_2$ h-DACs surface (TM=Ti, V, Cr and Fe); (b) Pt/Co@$Mo_2TiC_2O_2$ h-DACs surface; (c) Pt/Ni@$Mo_2TiC_2O_2$ h-DACs surface; (d) Pt/TM@$Mo_2TiC_2O_2$ h-DACs surface (TM = Cu, Zn).

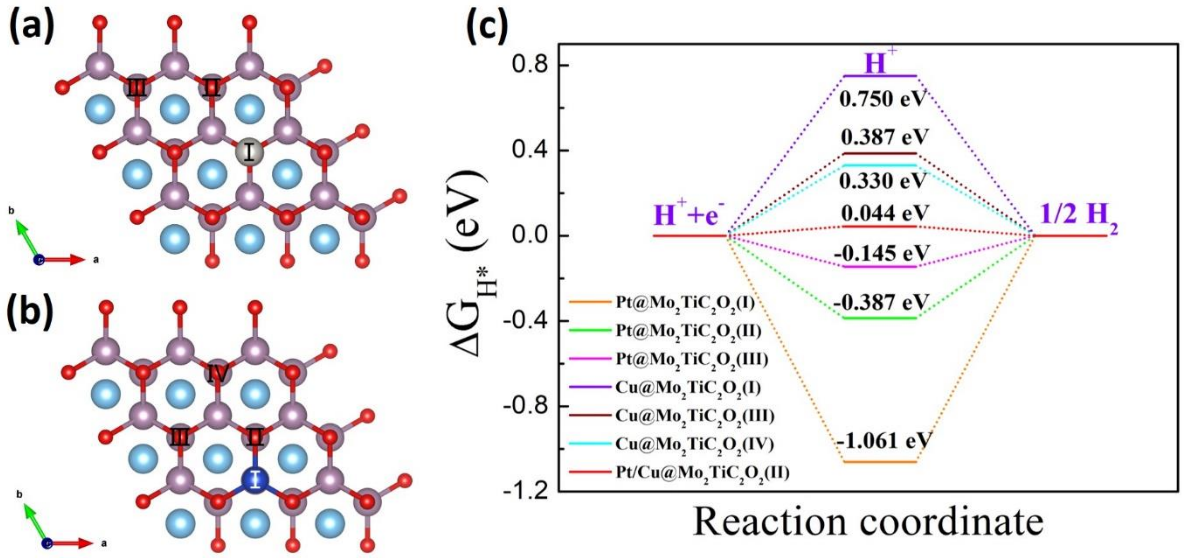

Figure S3. The possible H proton adsorption sites on the (a) Pt@$Mo_2TiC_2O_2$ SACs surface and (b) Cu@$Mo_2TiC_2O_2$ SACs. (c) Calculated Gibbs free energy profiles of HER for Pt@$Mo_2TiC_2O_2$ SACs, Cu@$Mo_2TiC_2O_2$ SACs and Pt/Cu@$Mo_2TiC_2O_2$ h-DACs.

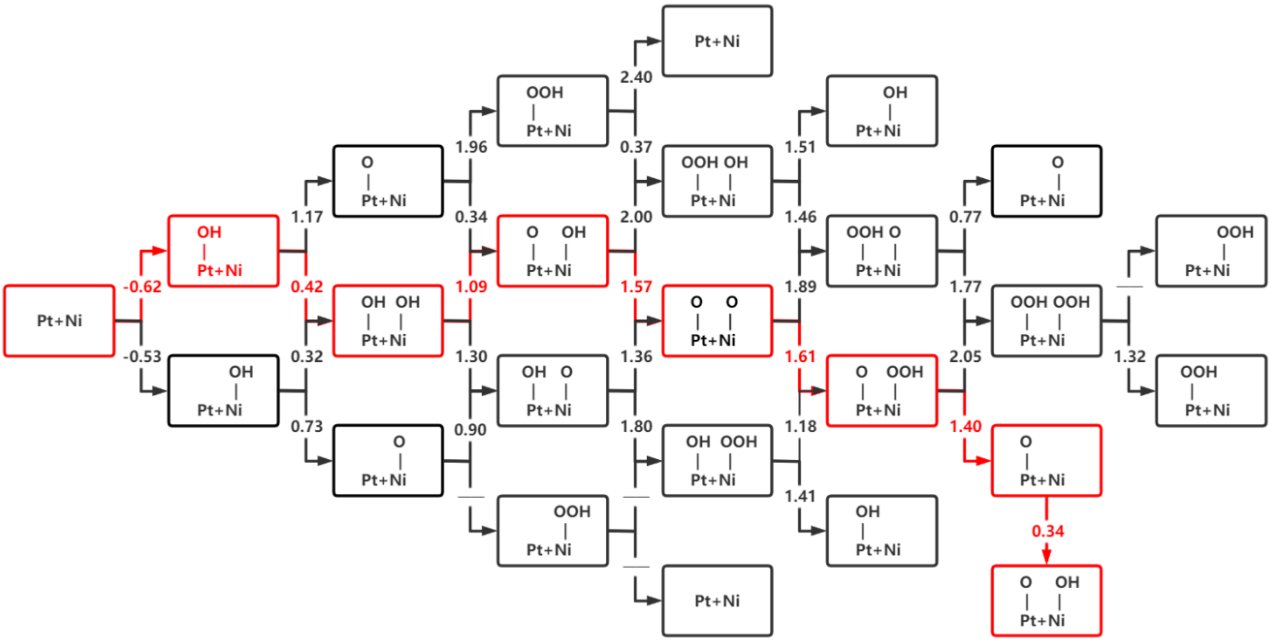

Figure S4. The diagram of all possible ORR pathways and corresponding Gibbs free energy on Pt/Ni@$Mo_2TiC_2O_2$ h-DACs, red lines represent the energy-favored reaction pathways.

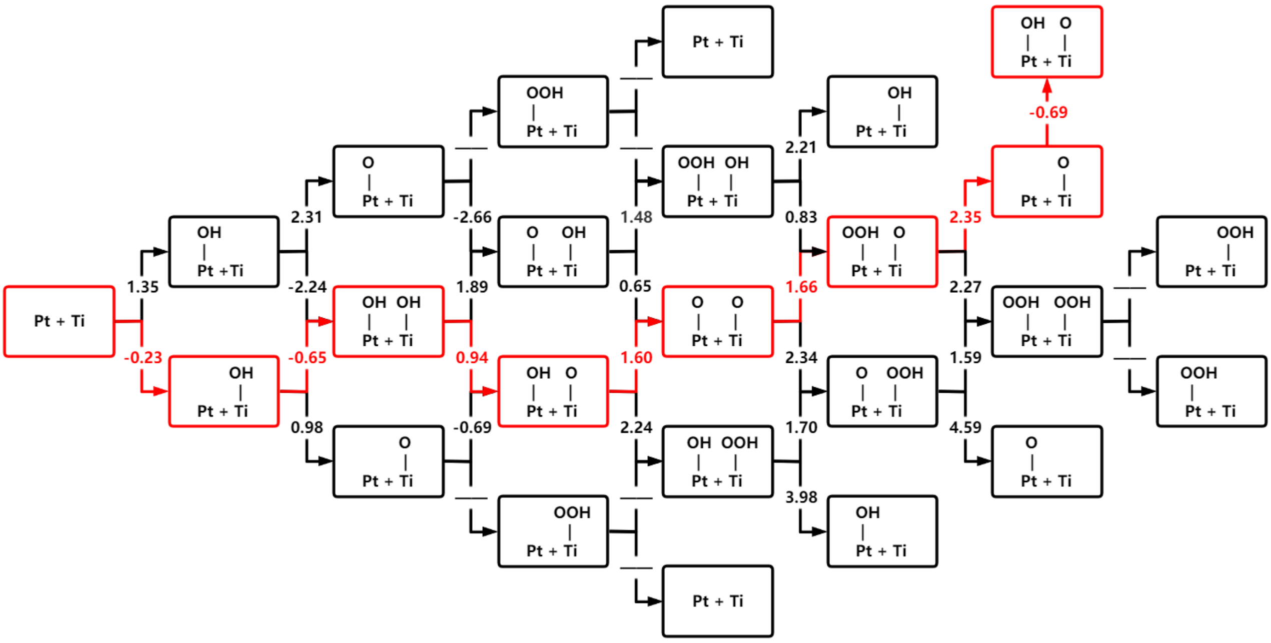

Figure S5. The diagram of all possible ORR pathways and corresponding Gibbs free energy on Pt/Ti@$Mo_2TiC_2O_2$ h-DACs, red lines represent the energy-favored reaction pathways.

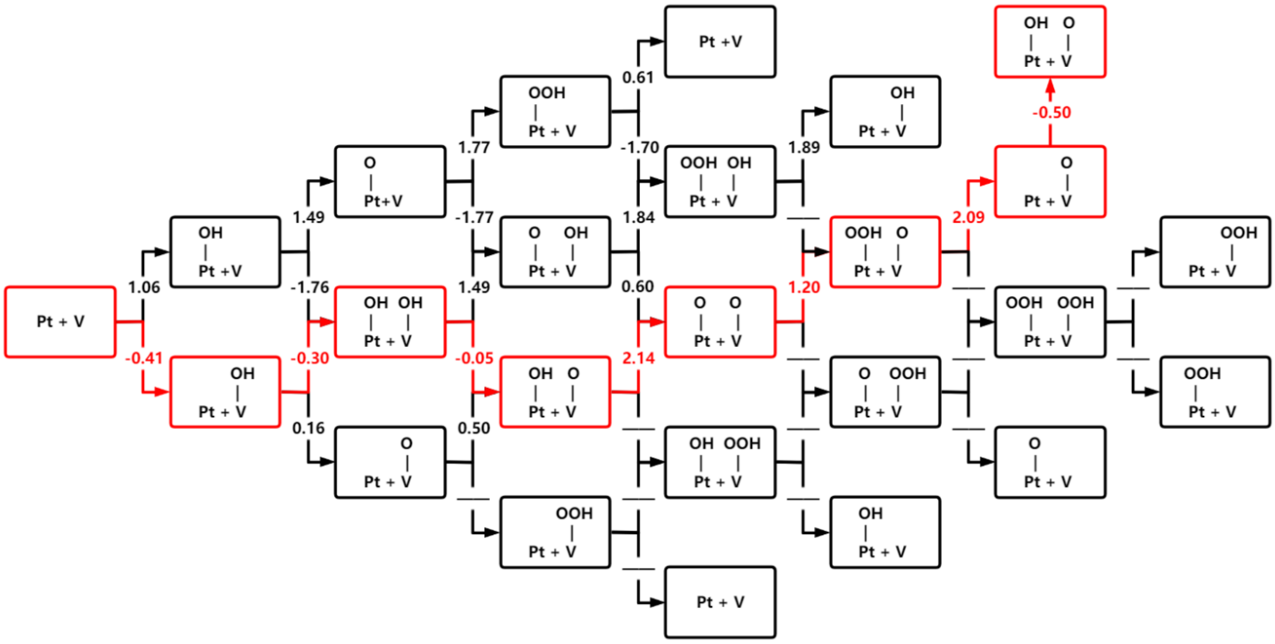

Figure S6. The diagram of all possible ORR pathways and corresponding Gibbs free energy on Pt/V@$Mo_2TiC_2O_2$ h-DACs, red lines represent the energy-favored reaction pathways.

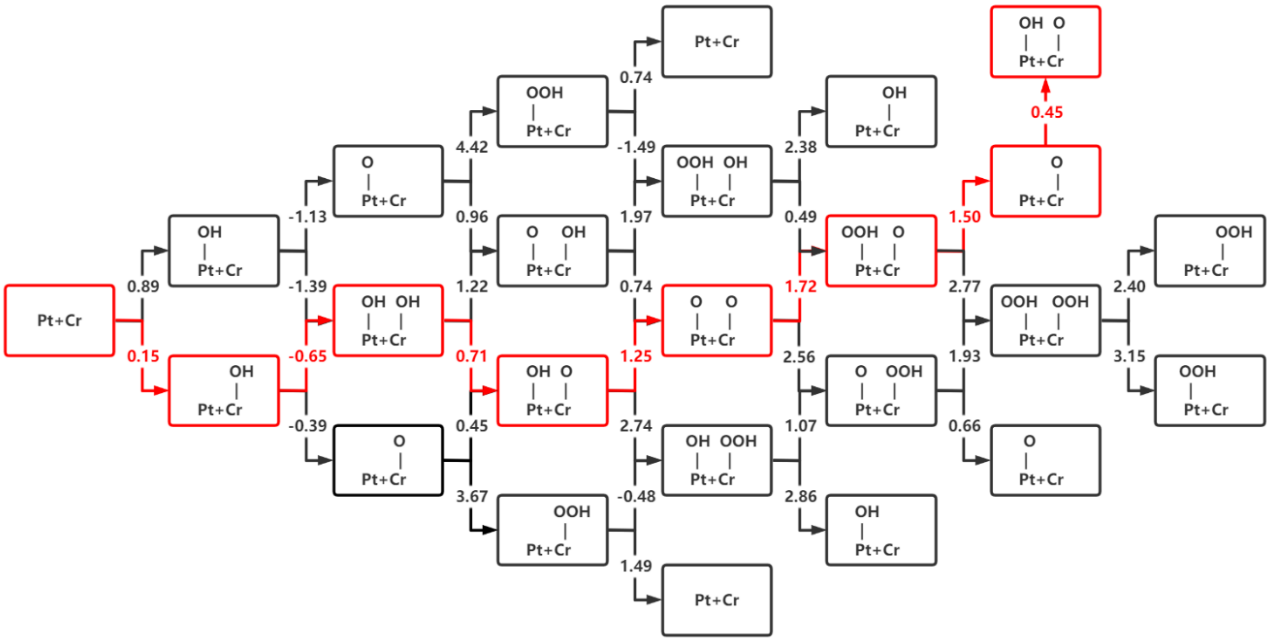

Figure S7. The diagram of all possible ORR pathways and corresponding Gibbs free energy on Pt/Cr@$Mo_2TiC_2O_2$ h-DACs, red lines represent the energy-favored reaction pathways.

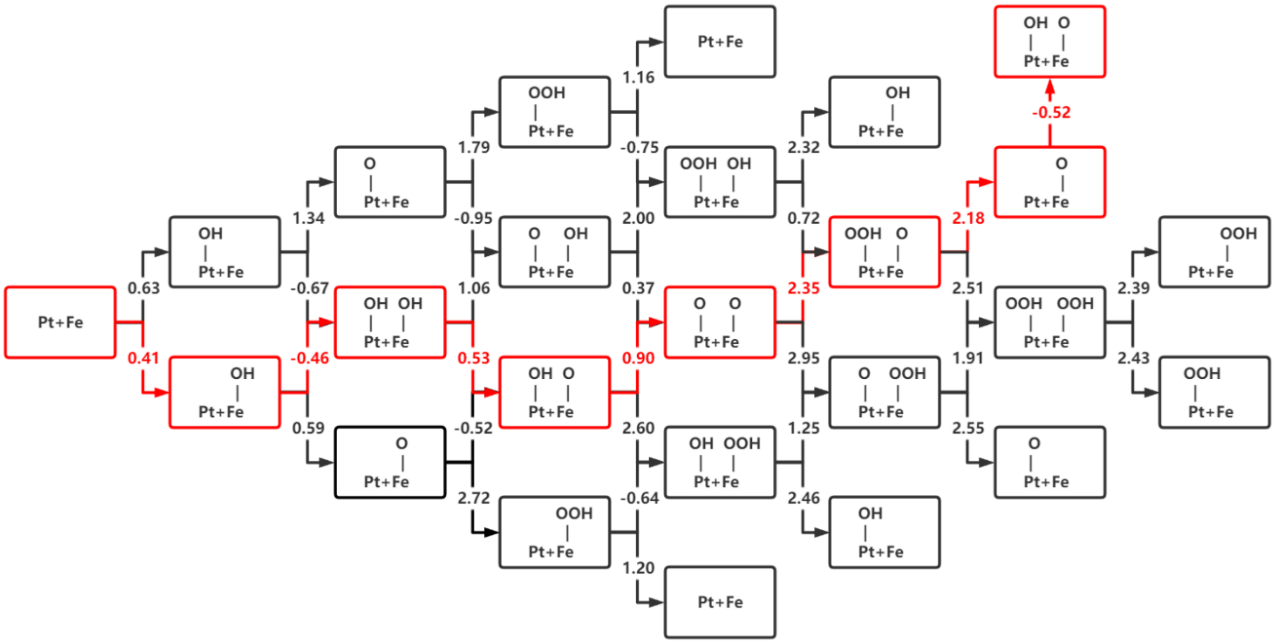

Figure S8. The diagram of all possible ORR pathways and corresponding Gibbs free energy on Pt/Fe@$Mo_2TiC_2O_2$ h-DACs, red lines represent the energy-favored reaction pathways.

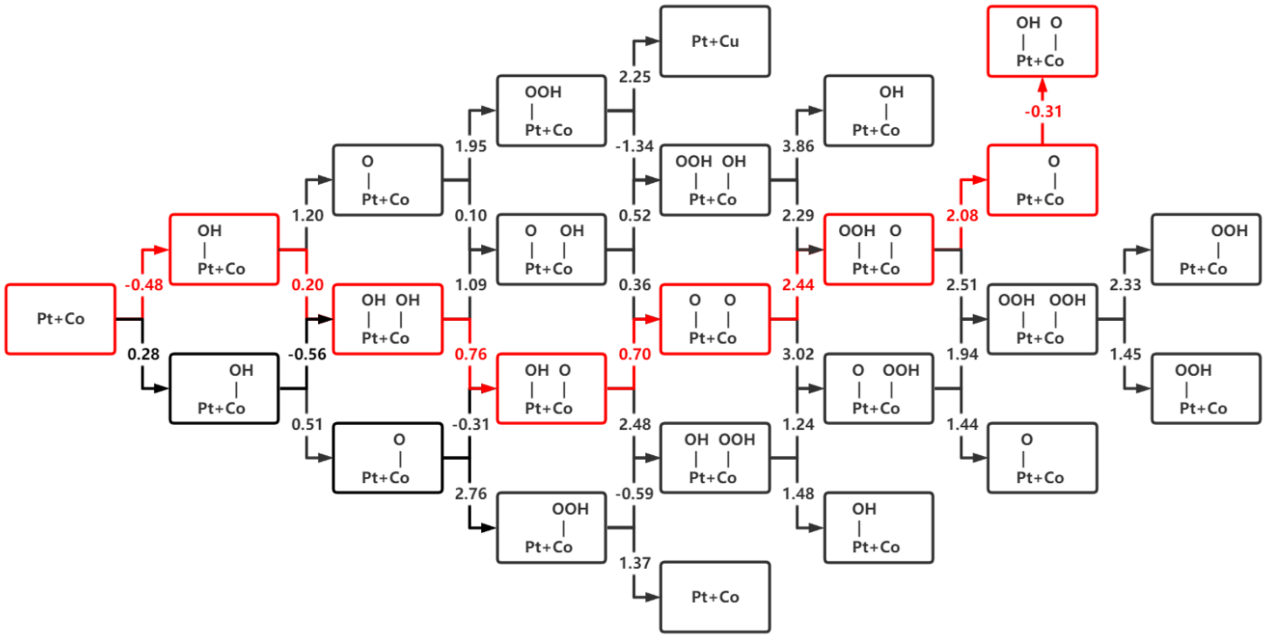

Figure S9. The diagram of all possible ORR pathways and corresponding Gibbs free energy on Pt/Co@$Mo_2TiC_2O_2$ h-DACs, red lines represent the energy-favored reaction pathways.

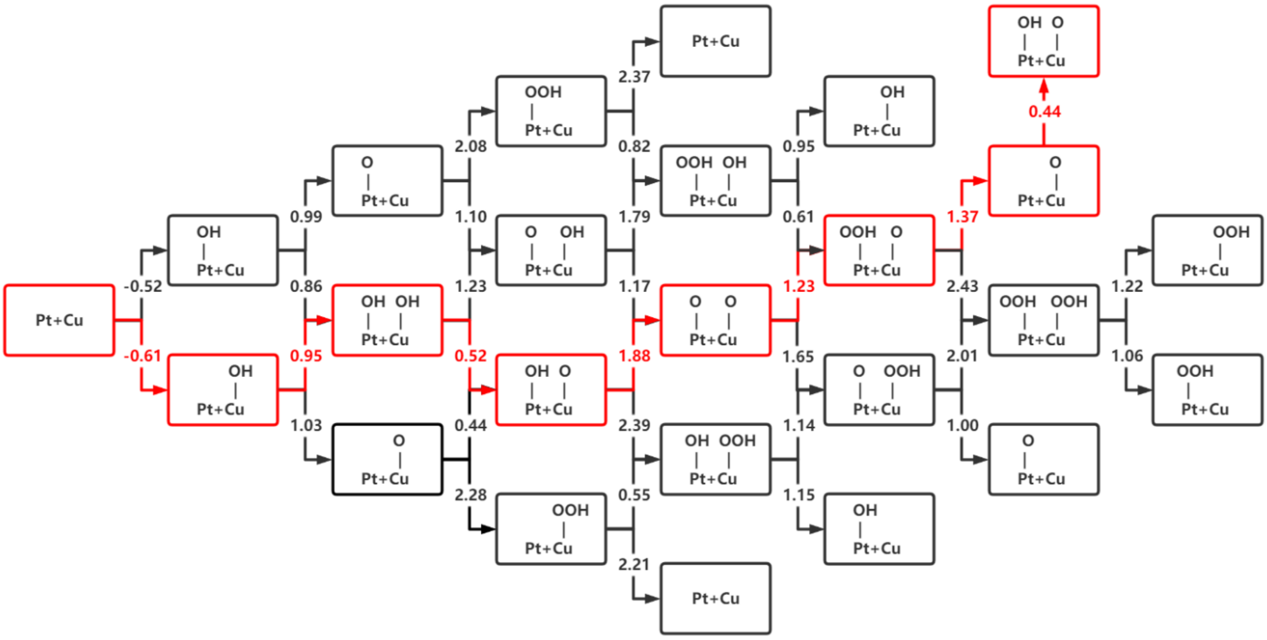

Figure S10. The diagram of all possible ORR pathways and corresponding Gibbs free energy on Pt/Cu@$Mo_2TiC_2O_2$ h-DACs, red lines represent the energy-favored reaction pathways.

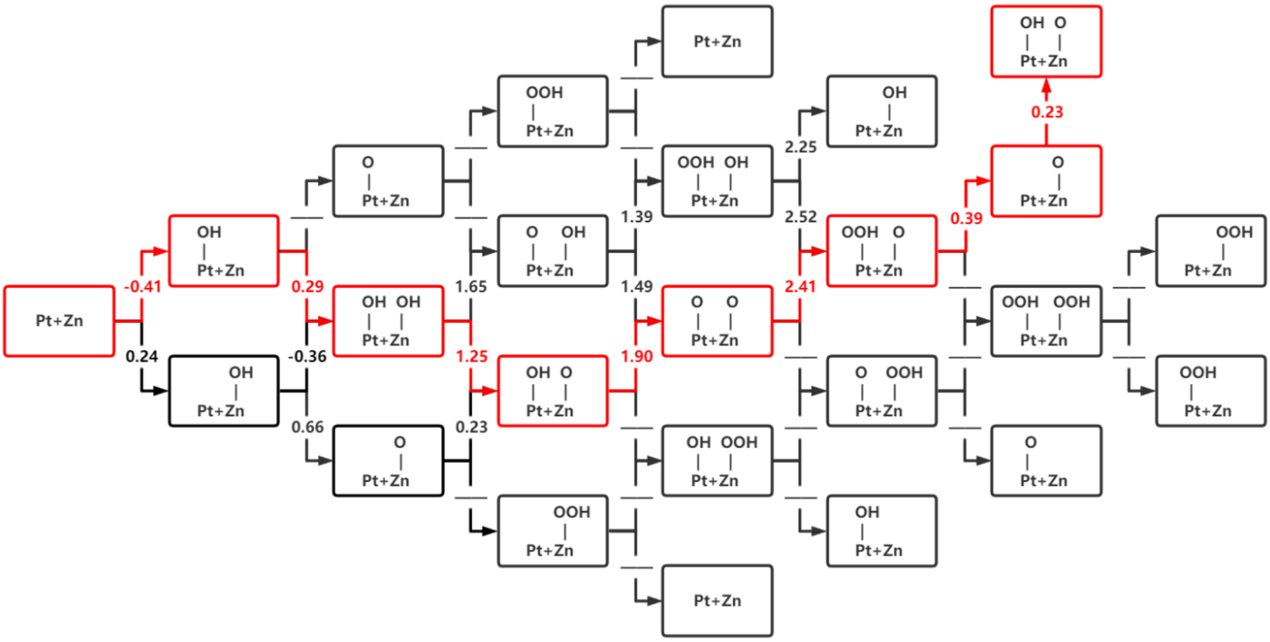

Figure S11. The diagram of all possible ORR pathways and corresponding Gibbs free energy on Pt/Zn@$Mo_2TiC_2O_2$ h-DACs, red lines represent the energy-favored reaction pathways.

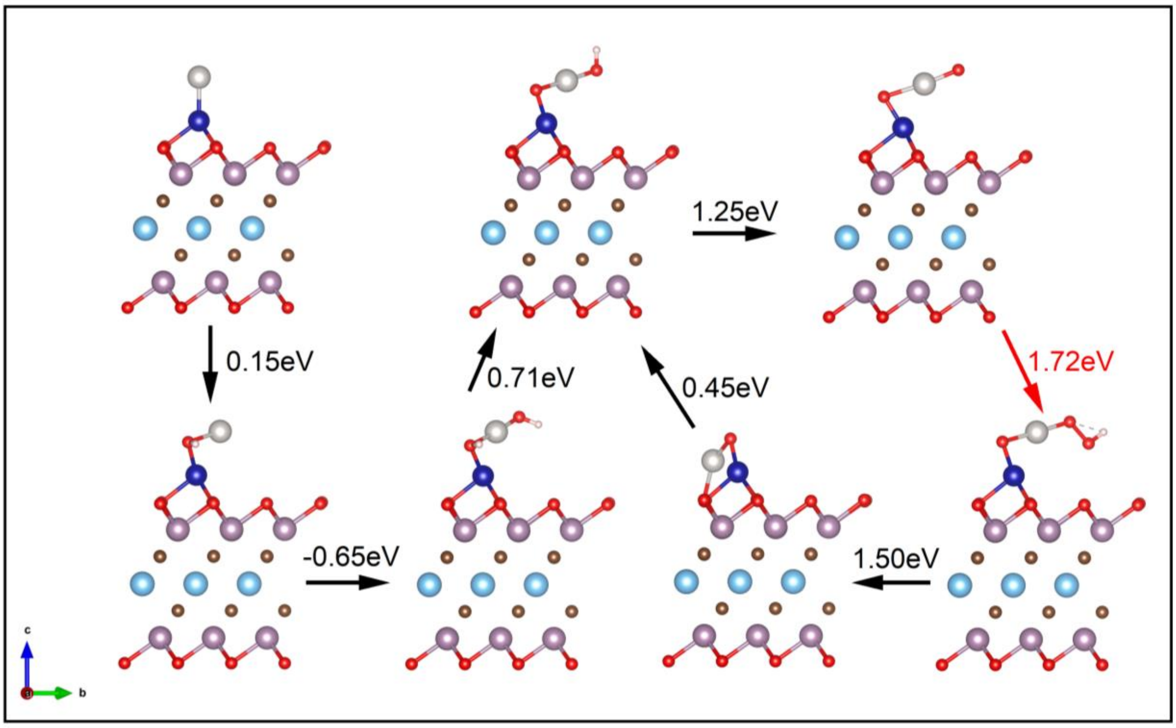

Figure S12. The configurations of oxygenated intermediates in energy-favored reactions pathways of OER on Pt/Cr@$Mo_2TiC_2O_2$ h-DACs. The red line represents the most energy-consuming step.

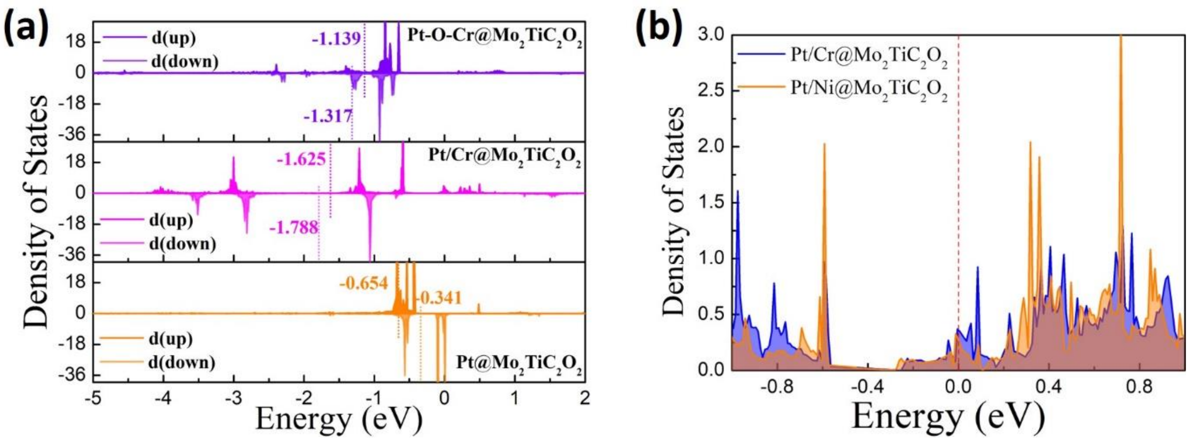

Figure S13. (a) The Projected DOS and the location d-band center of Pt atom for Pt@$Mo_2TiC_2O_2$, Pt/Cr@$Mo_2TiC_2O_2$ and Pt-O-Cr@$Mo_2TiC_2O_2$ h-DACs. (b) The electronic DOS (per atom) of Pt/Cr@$Mo_2TiC_2O_2$ h-DACs and Pt/Ni@$Mo_2TiC_2O_2$ h-DACs.

Table S1. ΔG_{H^*} of different H adsorption sites on the Pt/TM@$Mo_2TiC_2O_2$ DACs surface (TM = Ti, V, Cr, Fe, Co, Ni, Cu and Zn). Wherein, "x" represents structural instability after H proton adsorbed to the site. "-" represents the non-existent sites.

	Site I	Site II	Site III	Site IV	Site V	Site VI	Site VII
Pt/Ti@$Mo_2TiC_2O_2$	0.228	1.026	0.562	0.543	-	-	-
Pt/V@$Mo_2TiC_2O_2$	-0.187	×	0.298	0.301	-	-	-
Pt/Cr@$Mo_2TiC_2O_2$	-0.591	×	×	0.378	-	-	-
Pt/Fe@$Mo_2TiC_2O_2$	-0.724	×	×	0.214	-	-	-
Pt/Co@$Mo_2TiC_2O_2$	-0.813	×	×	0.159	-	-	-
Pt/Ni@$Mo_2TiC_2O_2$	-1.112	0.566	0.493	0.291	×	×	×
Pt/Cu@$Mo_2TiC_2O_2$	-0.706	0.044	0.268	0.354	×	×	-
Pt/Zn@$Mo_2TiC_2O_2$	-1.071	×	×	0.445	×	×	-

Table S2. The number of the charge transfer (Q_e, e) from Sigle-atom to substrate.

**Active center**	**Charge transfer (Q_e/e)**
$Ni@Mo_2TiC_2O_2$	-0.89
$Pt@Mo_2TiC_2O_2$	-0.45
$Pt/Ni@Mo_2TiC_2O_2$	-0.79
$O-Pt/Ni@Mo_2TiC_2O_2$	-0.76
$Pt/Cr@Mo_2TiC_2O_2$	0.38
$Pt-O-Cr@Mo_2TiC_2O_2$	-0.37
